# Impacts of spatio-temporal change of landscape patterns on habitat quality across Zayanderud Dam watershed in central Iran

**DOI:** 10.1038/s41598-024-59407-7

**Published:** 2024-04-16

**Authors:** Seyed Mohammad-reza Abolmaali, Mostafa Tarkesh, Seyed Alireza Mousavi, Hamidreza Karimzadeh, Saeid Pourmanafi, Sima Fakheran

**Affiliations:** Department of Natural Resources, Isfahan University of Technology, Isfahan, 84156-83111 Iran

**Keywords:** Environmental sciences, Biodiversity

## Abstract

The biodiversity of an ecosystem is greatly influenced by the spatio-temporal pattern of the landscape. Understanding how landscape type affects habitat quality (HQ) is important for maintaining environmental and ecological sustainability, preserving biodiversity, and guaranteeing ecological health. This research examined the relationship between the HQ and landscape pattern. The study presented an interpretation of the biodiversity variation associated with the landscape pattern in the Zayanderud Dam watershed area by integrating the Land Change Modeler and the InVEST model. Landsat images and maximum likelihood classification were used to analyze the spatio-temporal characteristics of the landscape pattern in 1991 and 2021. The future landscape pattern in 2051 was simulated using a Land Change Modeler. Subsequently, the InVEST model and the landscape maps were used to identify the spatial distribution of HQ and its changes over three periods. The mean values of the HQ in the study area were 0.601, 0.489, and 0.391, respectively, demonstrating a decreasing trend. The effect of landscape pattern change on HQ was also assessed based on landscape metrics, including PD, NP, SHDI, and CONTAG. HQ had a significant positive correlation with the CONTAG parameter (R = 0.78). Additionally, it had a significant inverse correlation with NP (R = − 0.83), PD (R = − 0.61), and SHDI (R = − 0.42). The results showed that the habitats in the northern region had lower quality compared to those in the southern parts of the Zayanderud Dam watershed. The density, diversity, and connectivity of landscape patches significantly influence the HQ in the study area. This research has the potential to enhance understanding of the impacts of land change patterns on biodiversity and establish a scientific basis for the conservation of natural habitats. Additionally, it can facilitate efficient decision-making and planning related to biodiversity conservation and landscape management.

## Introduction

As an important component of global change and a major driver, landscape patterns profoundly impact ecosystems and the services and goods they supply to humans^1^. The alteration of these patterns can lead to various natural effects and environmental operations^2^. Biodiversity is closely connected to the generation of ecosystem services (ES). Habitat quality (HQ), as an agent for biodiversity, represents the environmental capacity to supply suitable situations for population and person durability, extending from low to high, and resources accessible for survivorship, population persistence, and reproduction^3,4^. As well as being an important index of environmental health and security, which is vital for human well-being^5^. Landscape is the place of habitat, and land use change (LUC) could be an essential sign of the effect of human activities, containing conversions in the extent, intensity, and structure of land use and land cover (LU/LC). LUC vitally modifies HQ and the structure and composition of ecosystems, which influences nutrient cycling and energy flow among habitat patches^6^. The growth in LUC has driven habitat degradation, fragmentation, or even habitat loss, resulting in a continuous decrease in HQ^7^.

Land change models are excellent instruments for geographical, environmental, and other studies on LU/LC changes^8^. To study the past and predict future LULC changes in the watershed, a land change modeler (LCM) embedded in TerrSet has been used. The model is powerful because of its dynamic planning skills, appropriate calibrations, and ability to simulate several types of LU/LC^9^. LCM compares changes in LU/LC over several time periods, defines these changes, and presents the outcomes using multiple maps and graphs.

Regional changes in biodiversity and landscape patterns can be directly affected by changing habitat quality^10^. In order to assess ecosystem services functions, several models have been developed, such as the multiscale Integrated Ecosystem Services Model^11^, Artificial Intelligence for Ecosystem Services^12^, HQ model in InVEST (Integrated Valuation of Ecosystem Services and Tradeoffs model)^13^ and so on. The InVEST was expanded within the framework of the Natural Capital Project^14^. A partnership was established in 2007 with the University of Minnesota, Stanford University, and the World Wildlife Fund. The aim of this partnership is to develop a robust tool for valuing and quantifying various ecosystem services provided by landscapes. InVEST can exploit the advantages of little input data, precise outputs, and clear spatial projection to assess different ecosystem services such as biodiversity conservation, soil protection, and carbon reserves. Therefore, InVEST has been successfully used to evaluate ecosystem services in various areas^15^.

Lately, HQ research has focused mostly on a few countries^16^, and less research has been carried out in the semi-arid regions. In Iran, agricultural and industrial expansion and LU/LC change have led to a reduction in HQ^17^. Several studies in Iran have investigated the impact of LULC on HQ^18–20^. However, Limited studies in Iran have explored the relationship between landscape metrics and HQ^20–22^. These studies showed that HQ is closely associated with fragmentation resulting from landscape alterations and land use categories, thereby aiding in the comprehension of ecological attributes and habitat degradation^18,21,22^. Nevertheless, the lack of research in the central regions of Iran is apparent.

LCM and InVEST have demonstrated effective results, but there is limited research on integrating these two models. We can provide useful data to policymakers, managers, and planners to conserve biodiversity and achieve other landscape objectives by assessing the results of LCM and InVEST. The Zayanderud Dam watershed in central Iran is an important part of the Gavkhooni watershed. Rapid economic developments have led to a dramatic change in LU/LC types and environmental problems. The destruction of natural forests and rangelands has been driven by economic growth, mainly related to the expansion of agricultural land and build-up development. LUC in the Zayanderud Dam watershed has an incredibly damaging effect on natural forests and rangelands, as well as on the expansion of agricultural and construction areas^23^. The study has concentrated only on the spatial distribution of habitat quality due to time and cost constraints. The objectives of this research were to: (1) assess regional spatiotemporal changes of HQ by analyzing the LUC of the Zayanderud dam watershed over the past 30 years (1991), present, and the next 30 years (2051). (2) Determine the relationship between spatiotemporal variation in landscape characteristics and the HQ in the study area. (3) Summarize information to optimize planning and improve the LU/LC pattern.

## Methods

### Study area

**Figure 1:**
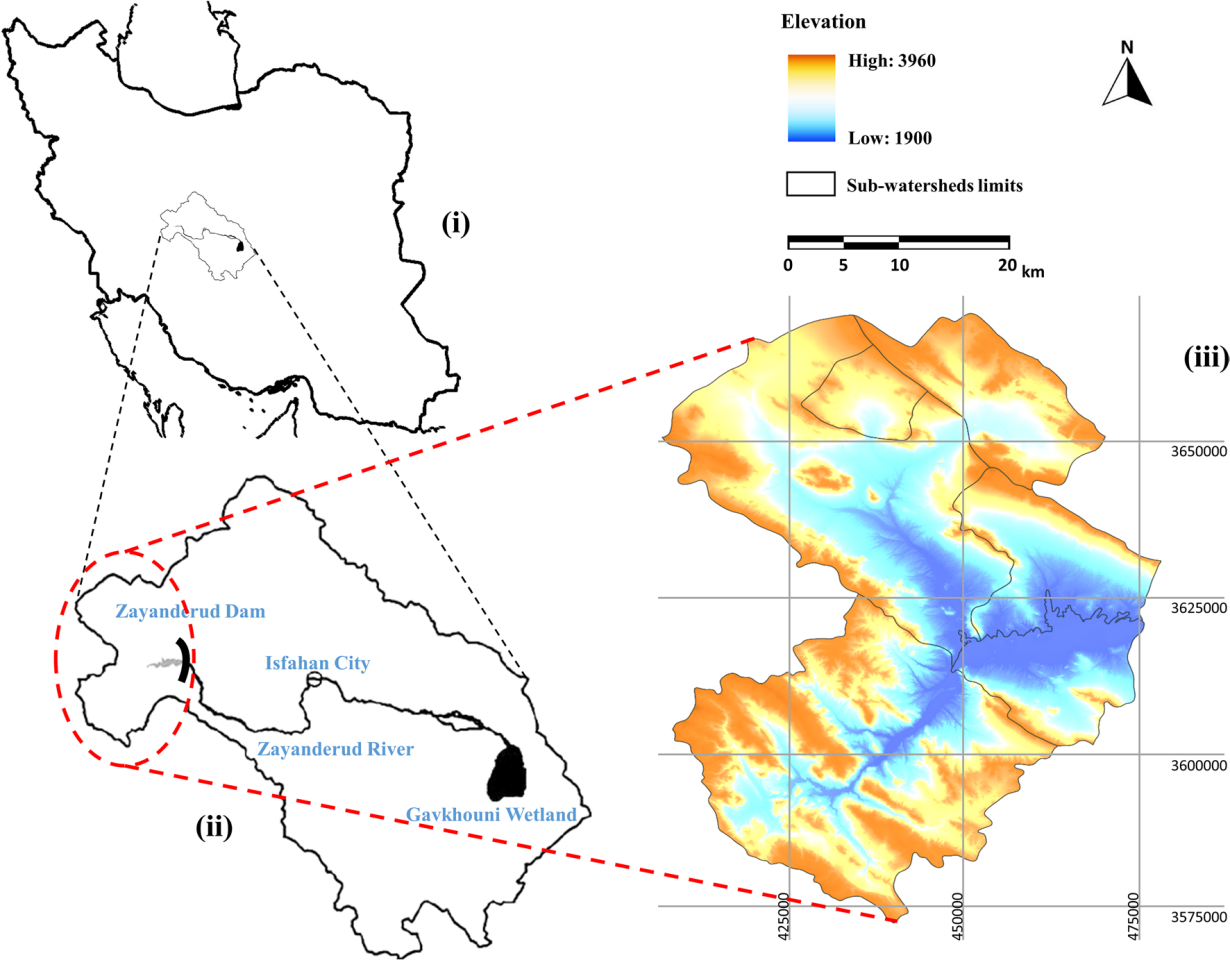
Location of the study area: (i) the Gavkhouni basin in Iran, (ii) the study area within the Gavkhouni basin, and (iii) sub-watersheds of the Zayanderud dam watershed, along with the Digital Elevation Model (DEM). This map was generated using ArcGIS 10.5 software (URL: https://www.esri.com/en-us/arcgis/products/index).

The study area was the Zayanderud Dam watershed located in the western part of the Gavkhouni watershed in central Iran, covering nearly 413,000 hectares. Zayanderud is the main river in the Gavkhooni watershed basin, which flows into the Gavkhooni wetland in the east. The Zayanderud Dam watershed gives the highest part of water yield. The average temperature of the basin is 8–13 °C with 350–1250 mm precipitation^23^. This region used to have a significant capacity for livestock grazing, with its primary LU/LC being forests and rangelands. Animal husbandry and agriculture are the primary sources of subsistence in these areas. However, in recent times, the transformation of rangelands into dry farming, intensive grazing, and urban expansion has led to the destruction of natural rangelands and forests in the area^24^. The area is the habitat of valuable and medicinal plant species such as *Quercus brantii*, *Kelussia odoratissima,* *Rheum ribes,* *Astragalus cyclophyllon, *and *Daphne mucronata*. The transformation of natural habitats into agricultural lands, along with the expansion of residential areas, has caused the destruction of forests and rangelands^25^. Figure 1 demonstrates the location of the Zayanderud Dam watershed in central Iran.

### LU/LC assessment

Landsat image classification is useful for automatically categorizing all pixels into LU/LC classes, facilitating the extraction of basic information^26^. For image classification, field studies were conducted in June 2021 to gather training areas for each LU/LC class to be used in the image classification process. Locations of each LU/LC were determined with GPS. Sample data were collected in homogeneous areas of LU/LCs regarding the spatial resolution of the images (30 m). The supervised classification was implemented using the maximum likelihood classification "MLC" method to produce a classification map after preprocessing Landsat images. The MLC method is a widely used algorithm for the classification of supervised satellite images^27^. Reference data collected from field studies were used for classifying 1991 and 2021 images (60 training areas were used for each LU/LC class in the image classification/420 training areas in total). Locations of each LU/LC were determined with GPS. Sample data were collected in the homogeneous areas of LU/LCs regarding the spatial resolution of the images (30 m). In this study, seven separable LU/LC are considered (Table 1). Description of Landsat images used in this study is provided in Table 2. The Normalized Difference Vegetation Index (NDVI) helped investigate the condition and density of the rangelands on a spatial basis^28,29^. Three rangeland condition classes were characterized based on the NDVI confidence interval (95%) and were classified as good (> 0.17), fair (0.12–0.17), or poor (0.01–0.12)^28^.

**Table 1:** Class description based on maximum likelihood classification.

Class name	Description
Zagros forest	Include the areas where trees mainly grow naturally
Rangeland/good	Include areas covered with natural vegetation that are used for grazing/ NDVI > 0.17
Rangeland/fair	Include areas covered with natural vegetation that are used for grazing/NDVI = 0.12–0.17
Rangeland/poor	Include areas that are poorly covered by natural vegetation/NDVI = 0.01–0.12
Water bodies	Water covers areas such as rivers and dam lakes
Agriculture	Includes all farms and gardens
Build up area	Includes all residential and man-made areas (cities, industrial areas, villages…)

**Table 2:** Description of Landsat images used in this study (*Landsat images Source: https://earthexplorer.usgs.gov/).

Dataset	Sensor	Path/Row	Date	Band Number	Resolution panchromatic
Landsat 5^*^	TM	164/36, 164/37	6.18.1991 7.2.1991	1, 2, 3, 4, 5	30
Landsat 8^*^	OLI	164/36, 164/37	6.14.2021 6.27.2021	2, 3, 4, 5	30

Some areas were selected as samples based on field study results for accuracy assessment and were used for Landsat images. The overall accuracy and Kappa coefficient were also determined^30,31^ (Eqs. (1) and (2))

1 $$Kappa\left(k\right)= \frac{{P}_{0}-{P}_{e}}{1-{P}_{e}}$$

where *P*_*0*_ represents the actual adaptation between two maps, and *P*_*e*_ demonstrates the probability of adaptation

2 $$\mathrm{Overall \; Accuracy}= \frac{\mathrm{ Number \; of \; Correctly\; Classified \;Pixels}}{\mathrm{Total \; Number\; of \;Pixels}}$$

### LU/LC prediction using Land Change Modeler (LCM)

The LCM model is the module of land change, which is linked to IDRISI. Clark's Laboratory and International Conservation have worked together for many years to develop IDRISI. The problem of rapid LU/LC change in recent years is addressed in the LCM model. In estimating LUC, this model is one of the basic models. Through the current land use situation, LCM can predict future LU/LC situations and is a good reference for decision-makers to make plans and expand conservation policies^32^. The LCM defines the factors affecting future LU/LC change and how much LU/LC change took place among earlier and later LU/LC and then computes a relative value of changes. To predict future trends in the study area, changes in LU/LC values for 1991 and 2021 have been analyzed. Within the LCM module, it is possible to generate maps of transition potential based on each sub-model and associated explanatory variables in three different ways: a similarity-weighted instance-based machine learning tool, logistic regression, and multi-layer perceptron (MLP) neural network^33^. The performance of the MLP is higher when modeling the relationship between nonlinear land changes and explanatory variables. As well as, when models of many transition types are used, it is more dynamic and flexible than any other^33^.

The LCM has been widely used in various global research endeavors as an innovative model for identifying and representing LU/LC alterations across diverse applications^34,35^. In recent studies, LCM has been used in central Iran to obtain accurate results^36–38^. This module incorporates historical data up to the present, enabling it to predict LU/LC changes effectively^39^. The process of forecasting future land cover within the LCM framework involves four primary stages: (1) examining past LU/LC modifications, (2) developing transition matrix maps, (3) validating the model, and (4) projecting future LU/LC maps.

This study identified descriptive variables that impact changes in LU/LC in the future using Cramer's V coefficient. This coefficient measures the correlation between dependent and independent variables, ranging from zero to one. Values closer to zero indicate a weak correlation, while values closer to one indicate a strong correlation. For a variable to be considered influential, the coefficient should be above 0.15^40^. The Cramer's V correlation coefficients for the descriptive variables (elevation, slope, distance to water bodies and rivers, distances to agricultural land, and distances to built-up areas) are presented in Table 3.

**Table 3:** The Cramer's V coefficients for the descriptive variables that impact LU/LC change.

Variables	Cramer's V coefficients
Elevation	0.434
Slope	0.258
Distance to water bodies and rivers	0.358
Distances to agricultural land	0.512
Distances to built-up areas	0.225

### HQ computation and prediction

The HQ model in InVEST projects the HQ for a preservation goal^14^. In order to determine what landscape constitutes habitat for different species, landscape maps are transformed into HQ maps^41^. The type of landscape determines the quality of the habitat in a cell, the landscape in circumambient cells, and the sensitivity of the habitat in the cell to the threats located near the landscape^42^. The HQ in a grid cell can be affected by the landscape around it. Each grid cell is given a HQ score of 0 to 1, with non-habitat scored as 0, and the highest HQ scored as 1^15^.

The source of degradation could be regarded as the types of landscape that are modified by humans, such as cities, agriculture, and roads, which cause edge effects^15^. Edge effect refers to changes in the physical and biological characteristics at a boundary between neighboring patches. The sensitivity of each habitat type to threats is the general aim of conserving landscape and ecology^43^. The HQ was computed by combining LU/LC classes and biodiversity threats. The HQ equation is demonstrated by Eq. (3):

3 $${Q}_{xj}={H}_{j}\left(1-\left(\frac{{D}_{xj}^{z}}{{D}_{xj}^{z}+{K}^{z}}\right)\right)$$

where Q_xj_ explains the HQ of x cell with land type j, H_j_ is the HQ of land type j, and K shows the half-saturation constant^14^.

4 $${D}_{xj}=\sum_{r=1}^{R}\sum_{y=1}^{{Y}_{r}}\left(\frac{{\omega }_{r}}{\sum_{r=1}^{R}{\omega }_{r}}\right){r}_{y}{i}_{rxy}{\beta }_{x}{S}_{jr}$$

5 $${i}_{rxy}=1-\left(\frac{{d}_{xy}}{{d}_{rmax}}\right)({\text{Linear}})$$

6 $${i}_{rxy}=exp\left(-\frac{{2.99d}_{xy}}{{d}_{rmax}}\right)({\text{Exponential}})$$

In Eqs. (4), (5) and (6), D_xj_ defines the total threat level in cell x with land type j, r is the number of threat factors, y denotes the set of cells on r’s raster map, w_r_ indicates the impact weight of threat r, i_rxy_ defines the degradation decay function through distance, β_x_ means the available grid cell x; S_jr_ shows the relative sensitivity of land type j to threat factor r, d_xy_ demonstrates the distance among pixel y and pixel x; d_rmax_ denotes the maximum impact threat distance of r originated in pixel y^14^.

Based on the literature review and expert knowledge, HQ of land type j and threat parameters were initially determined^15,44^. The values of model parameters have been proposed by 15 experts with a wide range of environmental experience, including expertise in experimental ecology, ecological modeling, environmental impact assessment, and knowledge of the study area^10,18–20^. The structure and meaning of the table that they should fill in and the parameters were described in detail before an expert score was performed, along with an explanation of how the InVEST HQ model functions.

The following parameters have been added to the model, including LU/LC maps (1991, 2021, and 2051), threat factors layers (including agriculture land, livestock grazing, mining land, main roads, minor roads, rural land and urban land), weight, the maximum effective distance of threat factors and decay (Table 4), sensitivity of landscape types to each threat (Table 5), and half-saturation constant. The HQ maps were created after running the InVEST-HQ model, with a pixel size set to 30 m based on Landsat images.

**Table 4:** Threat factors and maximum effective distances, weights, and type of decay in the study area.

Threat	Maximum effective distance (km)	Weight	DECAY
Agriculture land	8	0.82	Linear
Livestock grazing	6	0.72	Linear
Urban land	8	0.31	Exponential
Rural land	8	0.51	Exponential
Mining land	10	0.69	Exponential
Main roads	7	0.50	Linear
Minor roads	5	0.50	Linear

**Table 5:** LU/LC types and its sensitivity to each threat.

LU/LC	Habitat	Agriculture land	Livestock grazing	Urban land	Rural land	Mining land	Main roads	Minor roads
Zagros forest	1	0.70	0.61	0.74	0.70	0.80	0.85	0.70
Rangeland/good	1	0.75	0.82	0.68	0.70	0.81	0.85	0.70
Rangeland/fair	0.8	0.84	0.84	0.68	0.70	0.81	0.73	0.70
Rangeland/poor	0.6	0.84	0.71	0.54	0.70	0.80	0.73	0.70
Water bodies	0.5	0.40	0	0	0	0	0.35	0.35
Agriculture	0.2	0	0.30	0.70	0.75	0.85	0.50	0.65
Build up area	0	0	0	0	0	0	0.60	0.50

### The relationship between LU/LC pattern and HQ

**Figure 2:**
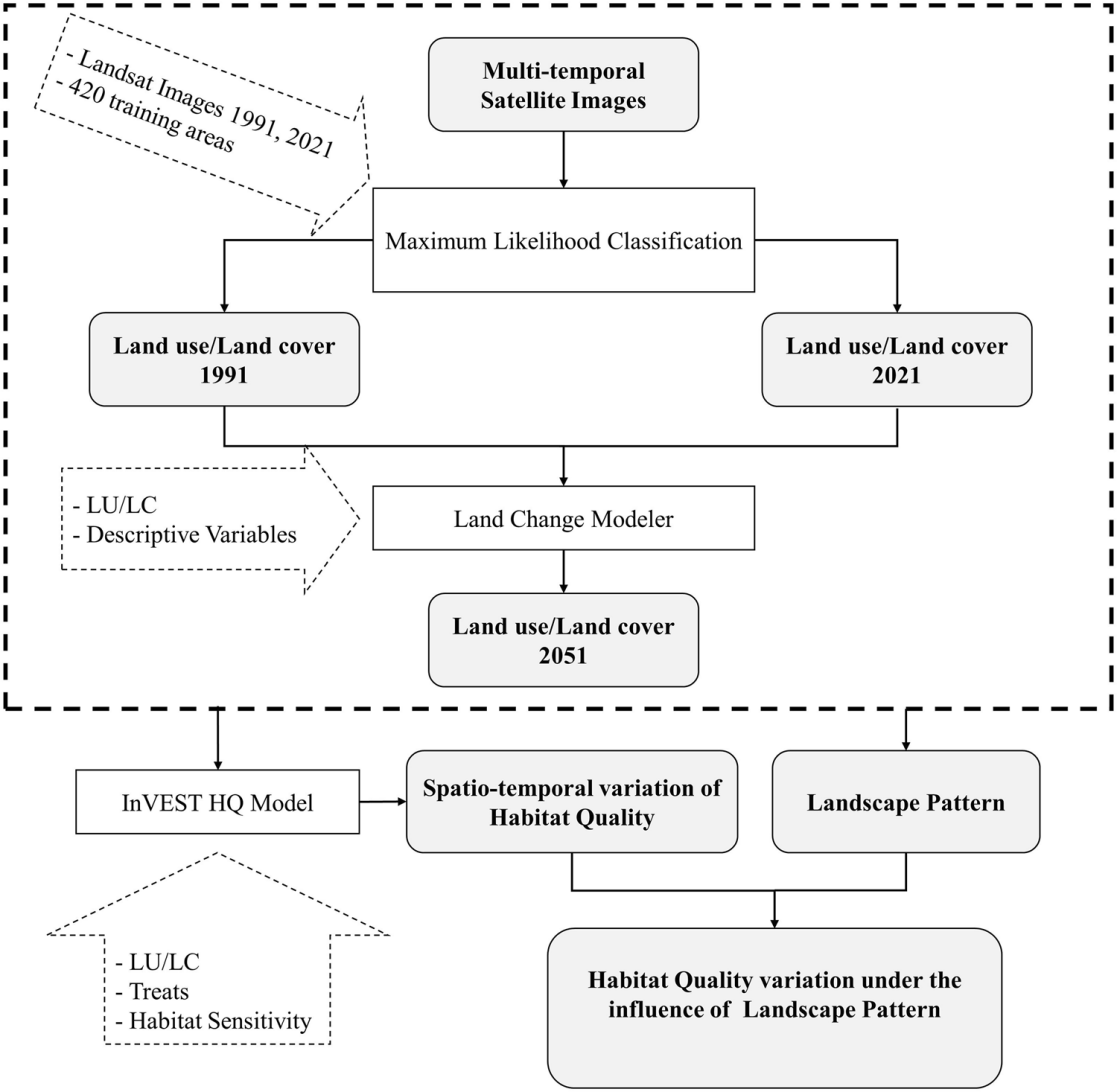
The workflow of the evolution of landscape patterns and habitat quality.

For measuring the spatial characteristics of LU/LC patterns, landscape metrics are appropriate tools. The relationship among landscape quantitative change, function, and structure can be described by metrics^45^. The impacts of different LU/LC on the environment and habitats can be considered according to landscape metrics. In this research, the impacts of LU/LC on HQ were recognized using patch density (PD), number of patches (NP), contagion index (CONTAG), and Shannon’s diversity index (SHDI). The selection of landscape metrics was based on the literature review in central Iran^20,22^. These metrics can determine the different characteristics of LU/LC in terms of its size, shape, composition, continuity, and fragmentation. The number and density of LULC patches in a given landscape are illustrated by the NP and PD indicators, respectively. Habitat fragmentation and discontinuity are reflected in the increasing values of these indicators. SHDI demonstrates the shifts in the ratio and number of land types. In a watershed, the richer land types, the higher SHDI value and the higher number of fragmented patches. The degree of agglomeration of the landscape is shown by the CONTAG Index; the higher CONTAG value, the higher the degree of agglomeration of the plaque and the better the linkage^45–47^. In FRAGSTATS, the metrics are calculated with LU/LC map at the landscape level, and Microsoft Excel 2016 was used to draw their charts. Figure 2 illustrates the research flow chart.

## Results

### Analysis of LU/LC change

**Figure 3:**
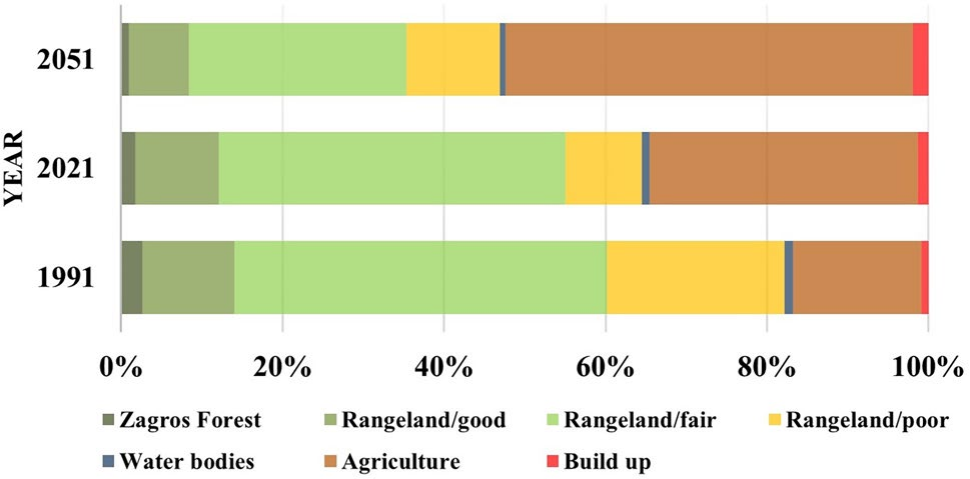
Charts indicate the percentage of each LU/LC from the total area in 1991, 2021 and 2051.

**Figure 4:**
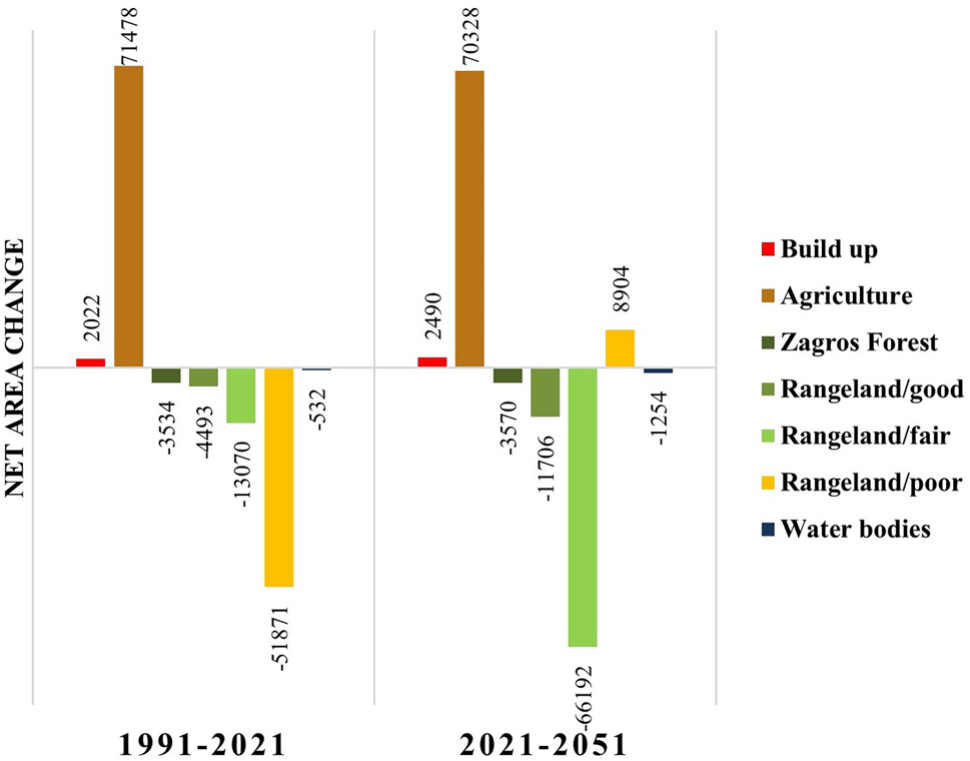
Charts indicate the net area change (ha) of each LULC in two period 1991–2021 and 2021–2051.

The present LU/LC map was created with an accuracy of 91% and a kappa coefficient of 0.88. The shift in LU/LC classes was calculated by measuring the net change using change analysis in LCM (see Supplementary Fig. S1 online). Habitat classes (forests and rangelands) covered 82%, 64%, and 45% of the total area in 1991, 2021, and 2051 respectively. The comparison of the spatio-temporal area of various LU/LC classes and the net area change of each LU/LC during the periods 1991, 2021, and 2051 was demonstrated in Figs. 3 and 4. The expanding agricultural and construction land areas can be attributed to the growing population and urban sprawl. The fair and poor rangelands seem to have transformed into low-yield agricultural lands.

### Spatiotemporal variation of HQ

**Figure 5:**
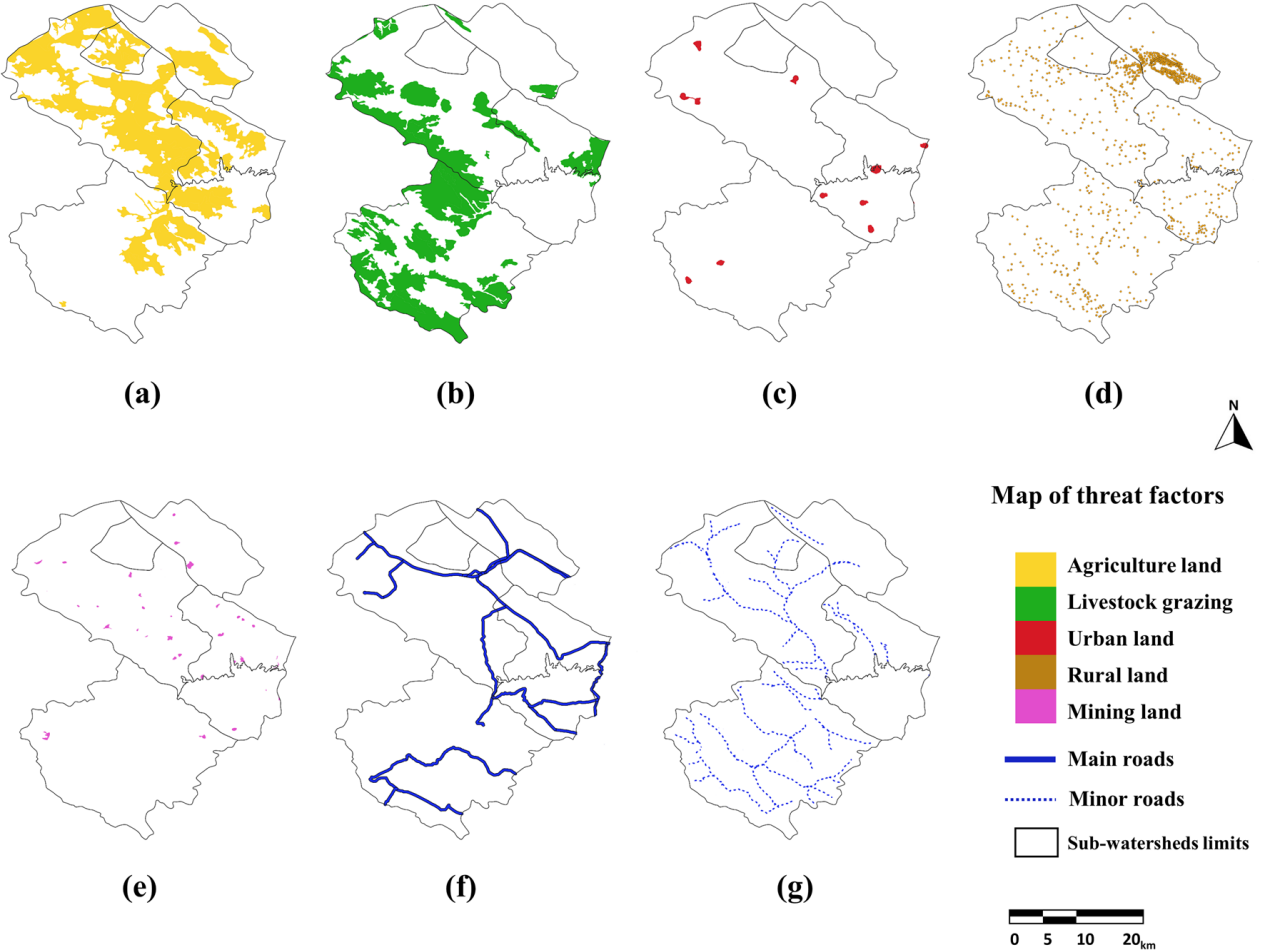
Threat factor maps: (**a**) agricultural land, (**b**) livestock grazing, (**c**) urban land, (**d**) rural land, (**e**) mining land, (**f**) main roads, (**g**) minor roads. This map was generated using ArcGIS 10.5 software (URL: https://www.esri.com/en-us/arcgis/products/index).

**Figure 6:**
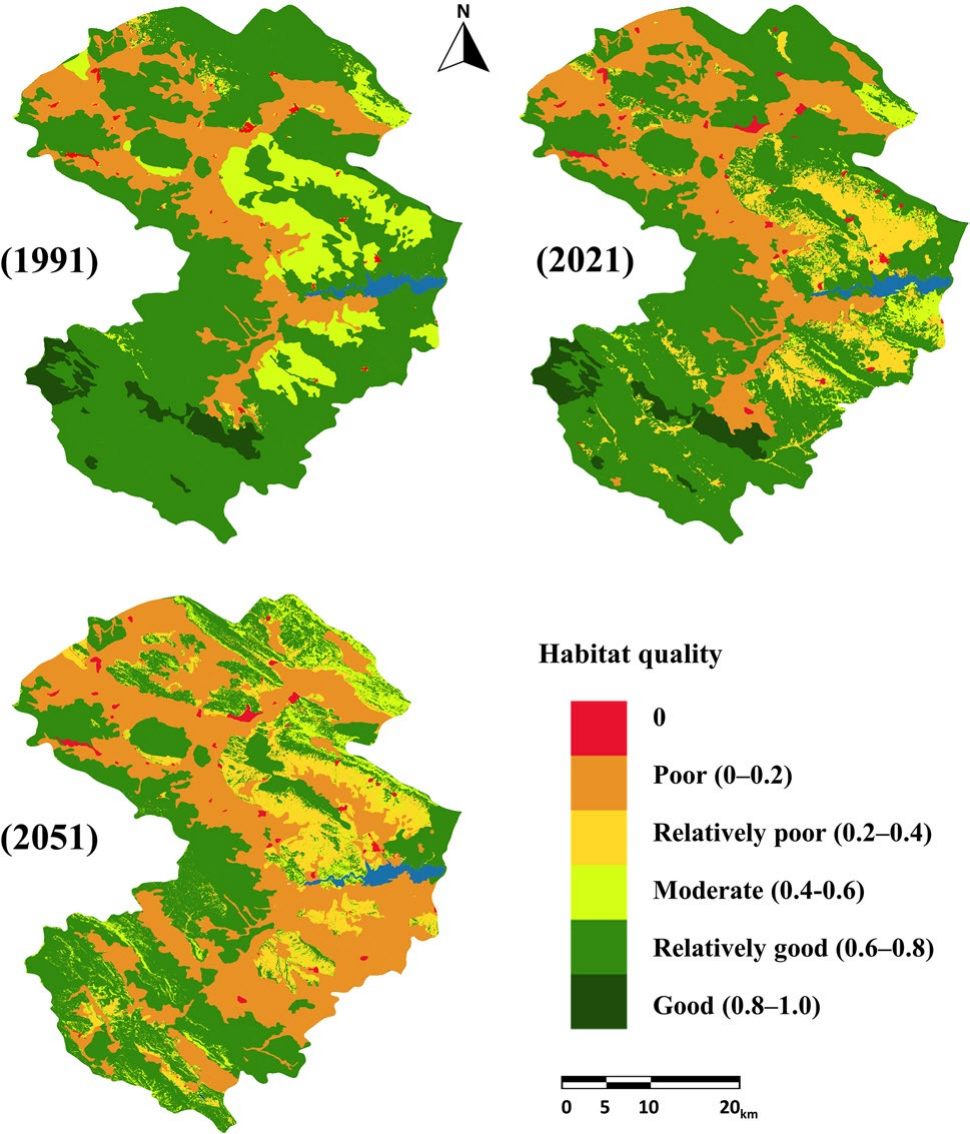
Habitat quality map in 1991, 2021 and, forecast map of habitat quality in 2051. This map was generated using ArcGIS 10.5 software (URL: https://www.esri.com/en-us/arcgis/products/index).

Threat factor maps (including agricultural land, livestock grazing, mining land, main roads, minor roads, rural land, and urban land) were generated using LU/LC maps. Figure 5 shows the maps of threat factors. The InVEST-HQ model generated HQ layers in various periods (Fig. 6). As summarized in Table 6, the HQ score was divided into six levels by the interval range: No habitat (0), Poor (0–0.2), Relatively poor (0.2–0.4), Moderate (0.4–0.6), Relatively good (0.6–0.8), and Good (0.8–1.0)^14,35^.

**Table 6:** Area of HQ classes in 1991, 2021 and 2051.

Habitat quality class	Area 1991 (ha)	Area 2021 (ha)	Area 2051 (ha)
0	7959	9981	12,471
0–0.2	65,741	101,057	209,121
0.2–0.4	20,578	138,298	102,510
0.4–0.6	190,717	52,985	48,623
0.6–0.8	117,051	101,258	40,252
0.8–1	10,945	9421	23

**Figure 7:**
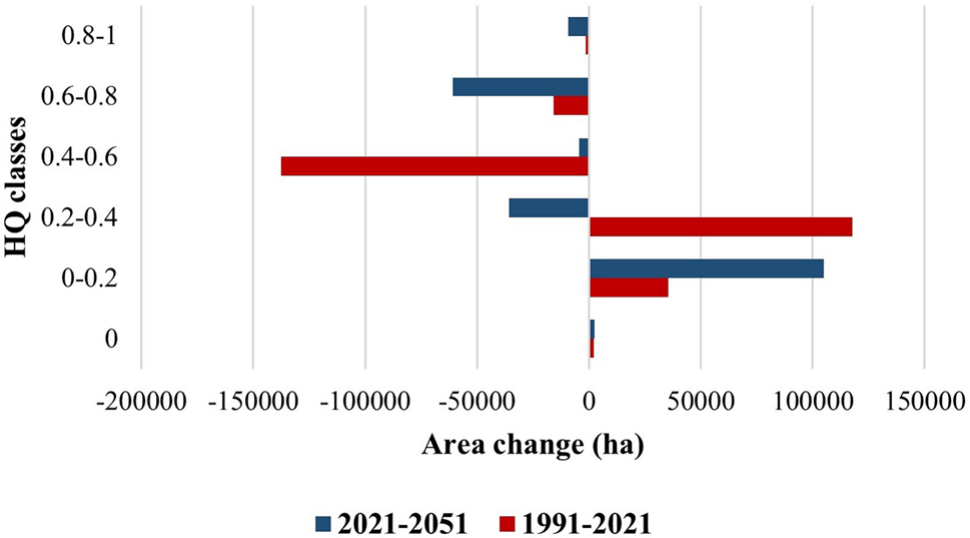
Net change area of HQ classes in two period 1991–2021 and 2021–2051.

Our research illustrated that the level of HQ in the Zayanderud Dam watershed reduced from 1991 to 2021. The InVEST model has been used to generate HQ maps under business-as-usual scenarios, and changes in HQ have been analyzed based on predicted LU/LC maps. The results demonstrated an overall decrease of 18.14% in the Zayanderud Dam watershed. Modelling predicted a significant decrease (19.55%) would be observed in 2021–2051, which was the same as the change in characteristics of LU/LC types. Figure 7 shows the net change in the area of HQ classes in two periods 1991–2021 and 2021–2051. Therefore, rapid population growth and LUC resulted in rapid degradation of the quality of its habitat. The average value of HQ in the Zayanderud Dam watershed was 0.601 in 1991, 0.489 in 2021, and 0.391 in 2051, showing a continuous decrease over the entire period.

The areas with low HQ scores were mostly located in the northern, central, and eastern parts of the Zayanderud Dam watershed, including Daran County and Chadegan County. Also, the areas with high HQ scores were mainly located in the south and west of the study area. The decreasing HQ will continue in future years under the current trend of LU/LC changes. With changes in landscape patterns and decreasing HQ, the landscape tends to become fragmented patches with reduced connectivity. As a result, it is vital to recognize and control human impact on landscape patterns and HQ.

## Discussion

The results indicated that in the southern and central parts of the study area, the most suitable and unsuitable habitats were found, respectively. Natural vegetation was one of the most important factors for increasing HQ in southern areas (Zagros forest and rangelands). Also, the factors contributing to improving the quality of these habitats include the distance from sources of threats and the limitation of access. Indeed, a factor that decreases the impacts of sources of degradation is habitat-to-threat source distance because a near threat to habitats reduces in terms of effect with increasing the distance. The LU/LC pattern showed that agricultural activities and built-up areas that caused HQ degradation dominate the study area's central and northern parts. The intensity and density of human destruction also influenced habitat and environmental degradation in the Zayanderud Dam watershed. These results show that the HQ of the study area has been substantially affected by LULC and its changes. Former research also refers to the effect of LU/LC change on HQ^17,20,48^.

As an indicator of biodiversity, The HQ in the InVEST model refers to the environment's ability to provide adequate conditions for population and individual durability^49^. The model considers higher biodiversity levels in areas with high HQ. Biodiversity is reduced after the destruction of similar habitats^14^. Nevertheless, there are not necessarily high levels of biodiversity in areas with good HQ. In addition, the origin of this model is more willing to use natural vegetation habitats, which have specific restrictions in the Zayanderud Dam watershed. Plus, it should be noted that the watershed has a dummy border where the threats to habitat instantly out of the research area border have been ignored and clipped. As a consequence, the threat intensity on the margins of a determined area will always be lower^10^. The HQ scores should be expounded as comparative scores with superior scores demonstrating that the landscape is more suitable for a certain conservation goal. It is not feasible to interpret the landscape HQ score as a forecast of species survival, landscape durability, or any other specific species preservation and conservation action. HQ measures are not converted into monetary values in the InVEST habitat model.

**Figure 8:**
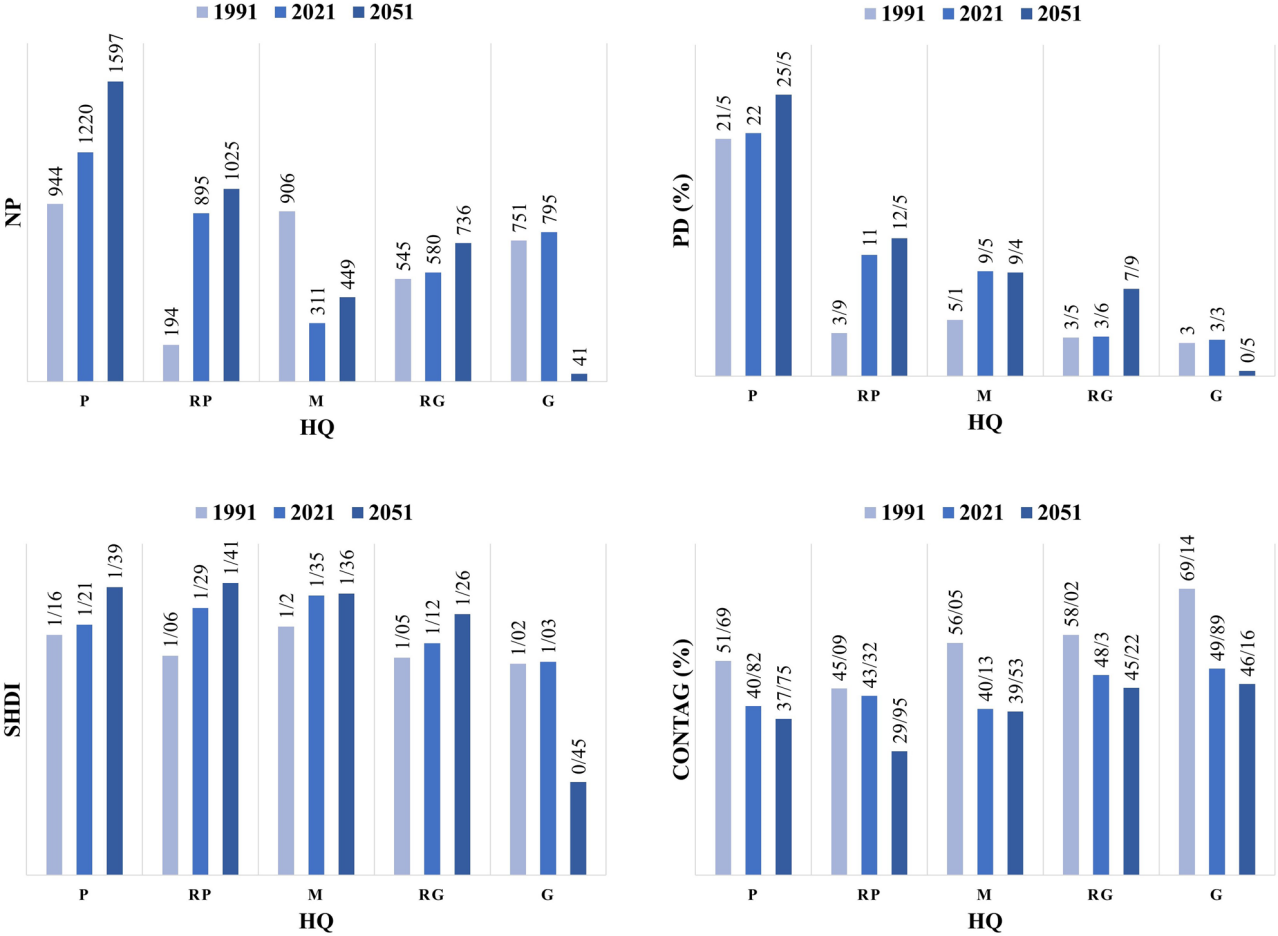
The trend of the landscape metrics based on the classes of habitat quality in 1991, 2021and 2051 (number of patches (NP), patch density (PD), contagion index (CONTAG), and Shannon’s diversity index (SHDI)).

Four landscape metrics have been calculated at the landscape level in this research (Table 7). The effect of landscape patterns on HQ was also evaluated using several landscape metrics, including PD, NP, CONTAG, and SHDI (Fig. 8). PD and ND indicators denote the rate of habitat fragmentation. CONTAG and SHDI define the connectivity and diversity of LU/LCs. PD, NP, and SHDI alterations indicate an additive trend in 2021 and 2051, whereas CONTAG changes demonstrate a decrease in 2021 and 2051. Generally, the tendency of all metrics showed that the HQ of the Zayanderud Dam watershed was reduced by increasing habitat fragmentation and diversity of LU/LCs and decreasing contagion and connectivity in 2021 and 2051.

**Table 7:** Landscape metrics in 1991, 2021 and 2051 at landscape level.

	NP	PD	CONTAG	SHDI
1991	3340	37.1	50.95	1.11
2021	3801	49.4	47.51	1.23
2051	3848	55.8	39.22	1.31

These results showed that the number, density, diversity, and connectivity between landscape patches and LU/LC type strongly impact the HQ of the study area. Indeed, two key factors significantly influencing HQs within the Zayanderud Dam watershed are spatial pattern and LU/LC type. Former researches also recognized LU/LC and landscape metrics as effective factors in environmental situations and processes^20,50–52^.

**Figure 9:**
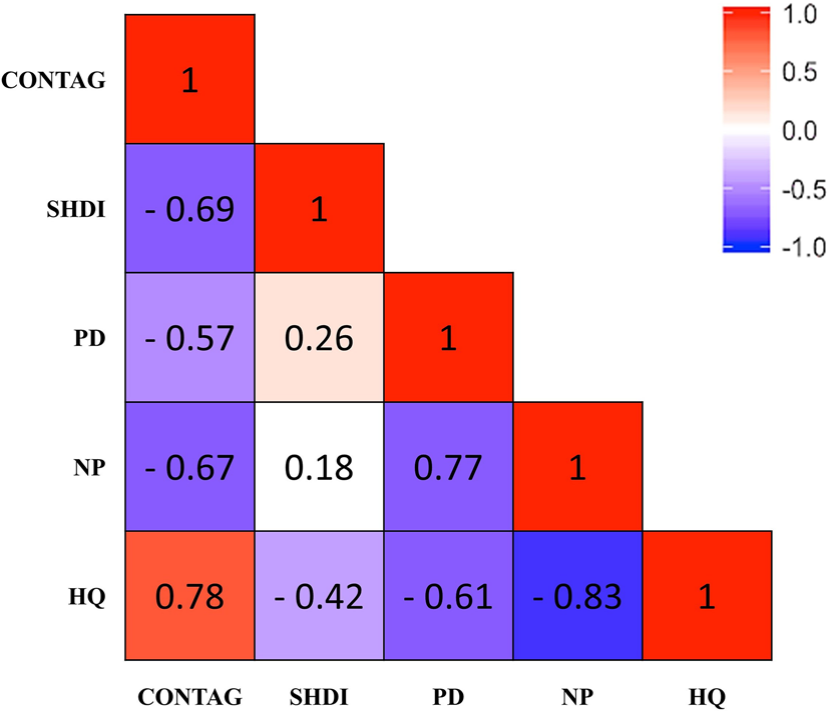
Results of Pearson correlation coefficient between the HQ and landscape metrics.

The linear correlation analysis between the HQ and landscape metrics showed that at the 0.05 significance level, HQ had a significant positive correlation with the CONTAG parameter (R = 0.78). Additionally, it had a significant inverse correlation with NP (R = -0.83), PD (R = -0.61), and SHDI (R = -0.42). Figure 9 illustrates Pearson's correlation coefficients and the correlation matrix between the HQ and landscape metrics. These dependencies show that landscape patterns are essential to the habitat conditions because landscape components are considered obstacles to expanding threats. Ahmadi Mirghaed and Souri^53^ also pointed out that the HQ is always influenced by landscape metrics such as NP, PD, LPI, and LSI. Chu et al.^10^ noted that changes in landscape patterns over time cause changes in HQ.

The relationship between HQ, LU/LC, and landscape metrics has been explored in this study. Furthermore, the importance of maintaining the HQ of the Zayanderud Dam watershed for stakeholders and regional decision-makers has been highlighted. Nevertheless, more data about ecosystem situations and ecological connections are available. The study of landscape patterns can provide an accurate and significant evaluation of ecosystems to recognize the areas with high HQ and degraded lands. In order to improve our understanding of the ecological condition in a region and make better decisions for land management, it has been confirmed that combining HQ with landscape metrics is an adequate indicator for assessing ecosystems^20^, as well as Zheng and Li^54^ reported that effective LU/LC planning based on habitat quality is essential for achieving sustainable regional development. About natural ecosystems such as rangelands and forests, decision-makers should recover the natural biodiversity system^55^. The vegetation conservation and restoration project should be carried out regarding particular circumstances of the area. Hence, to achieve sustainable development, effective and reasonable regional landscape planning is necessary.

Our study has limitations. First, the study area has a dummy border where the threats to the habitat just beyond the boundary have been disregarded. As a result, the level of threat intensity at the edges of the study area will be lower. Second, the HQ model requires several parameters. The relevant parameters utilized in this study were acquired from field studies, Landsat images, previous research, and expert knowledge. This could lead to uncertainty in the estimated results. Especially the LU/LC classification error can introduce skepticism in the HQ results, particularly in the future. Final, a method for validating the HQ score is not mentioned in the model manual or references^10,14,54^.

## Conclusions

The purpose of the current study was to evaluate the variation of landscape type and pattern, HQ, and investigate biodiversity in response to the impression of the LU/LC dynamics in the Zayanderud Dam watershed. The LCM model executed well in predicting future land types (2051) by incorporating geographical factors as restrictive factors. Over the past 30 years, the HQ has decreased. Future predicted HQ maps represented the same tendencies as the last 30 years, denoting the effect of land type dynamics on HQ and biodiversity. It is clear that population growth, accompanied by the increase in construction sites and low-yield agricultural lands in central areas of the Zayanderood Dam watershed basin, has resulted in biodiversity losses.

The transition from forest and rangeland to agricultural lands and residential areas is expected to affect HQ adversely. The key challenge lies in finding a balance between conserving areas with abundant HQ and meeting the demands of a growing population. To address this issue, we recommend that, First, local governments take steps to prevent LUC in areas with high HQ. Second, governments should promote policies supporting agricultural livelihood systems on agricultural lands. Third, Payment for Ecosystem Services (PES) projects are considered suitable for conserving high HQ areas.

The results proposed the feasibility of analyzing the impact of LU/LC changes and land type pattern dynamics on HQ. This research supplies a scientific foundation for optimizing regional conservation plans for natural areas, and a clear and efficient framework was offered for stakeholders, local government, and decision-makers to landscape administratorship and planning, sustainable development, and habitat conservation. In future studies, it is suggested to recognize the relationship between the economy and habitat quality, as well as the economic valuation of HQ in different years.

### Supplementary Information

Supplementary Figure S1.

## Data Availability

The datasets used and/or analyzed during the current study available from the corresponding author on reasonable request.
