# Supplementary material for: Impacts of spatio-temporal change of landscape patterns on habitat quality across Zayanderud Dam watershed in central Iran
Source: Sci Rep. 2024 Apr 16;14:8780. doi: 10.1038/s41598-024-59407-7 (PMC11021427; doi:10.1038/s41598-024-59407-7)
Supplement: Supplementary file 1 — Supplementary Figure S1. [file 41598_2024_59407_MOESM1_ESM.pdf]

Supplementary Information

Impacts of spatio-temporal change of landscape patterns on habitat quality across Zayanderud Dam watershed in central Iran

Seyed Mohammad-reza Abolmaali[1], Mostafa Tarkesh[1], Seyed Alireza Mousavi[1], Hamidreza Karimzadeh[1], Saeid Pourmanafi[1] and Sima Fakheran[1]

[1] Department of Natural Resources, Isfahan University of Technology, Isfahan 8415683111, Iran.

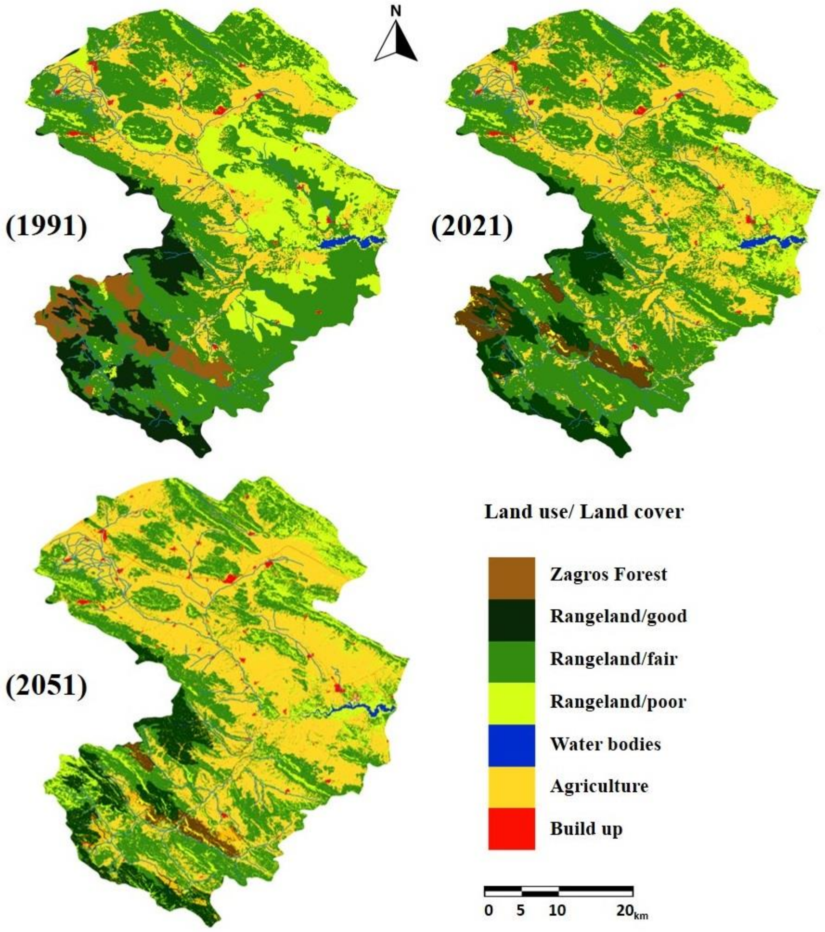

Supplementary Fig. S1 Land use/land cover map of the Zayanderud dam watershed basin in 1991, 2021 and 2051. This map was generated using ArcGIS 10.5 software (URL:https://www.esri.com/en-us/arcgis/products/index).
